# Rumen-protected methionine and lysine supplementation to the low protein diet improves animal growth through modulating colonic microbiome in lambs

**DOI:** 10.1186/s40104-025-01183-z

**Published:** 2025-03-18

**Authors:** Zhibin Luo, Huimin Ou, Zhiliang Tan, Jinzhen Jiao

**Affiliations:** State Key Laboratory of Forage Breeding-by-Design and Utilization, CAS Key Laboratory of Agroecological Processes in Subtropical Region, Institute of Subtropical Agriculture, Chinese Academy of Sciences, Changsha, Hunan 410125 China

**Keywords:** Amino acid metabolism, Colonic microbiome, Lamb, Metagenome, Rumen-protected amino acids

## Abstract

**Background:**

Dietary protein level and amino acid (AA) balance are crucial determinants of animal health and productivity. Supplementing rumen-protected AAs in low-protein diets was considered as an efficient strategy to improve the growth performance of ruminants. The colon serves as a crucial conduit for nutrient metabolism during rumen-protected methionine (RPMet) and rumen-protected lysine (RPLys) supplementation, however, it has been challenging to clarify which specific microbiota and their metabolites play a pivotal role in this process. Here, we applied metagenomic and metabolomic approaches to compare the characteristic microbiome and metabolic strategies in the colon of lambs fed a control diet (CON), a low-protein diet (LP) or a LP diet supplemented with RPMet and RPLys (LR).

**Results:**

The LP treatment decreased the average daily weight gain (ADG) in lambs, while the LR treatment tended to elicit a remission in ADG. The butyrate molar concentration was greater (*P* < 0.05), while acetate molar concentration (*P* < 0.05) was lower for lambs fed the LP and LR diets compared to those fed the CON diet. Moreover, the LP treatment remarkably decreased total AA concentration (*P* < 0.05), while LR treatment showed an improvement in the concentrations of methionine, lysine, leucine, glutamate, and tryptophan. Metagenomic insights proved that the microbial metabolic potentials referring to biosynthesis of volatile fatty acids (VFAs) and AAs in the colon were remarkably altered by three dietary treatments. Metagenomic binning identified distinct microbial markers for the CON group (*Alistipes* spp., *Phocaeicola* spp., and *Ruminococcus* spp.), LP group (*Fibrobacter* spp., *Prevotella* spp., *Ruminococcus* spp., and *Escherichia coli*), and LR group (*Akkermansia muciniphila* and RUG099 spp.).

**Conclusions:**

Our findings suggest that RPMet and RPLys supplementation to the low-protein diet could enhance the microbial biosynthesis of butyrate and amino acids, enriche the beneficial bacteria in the colon, and thereby improve the growth performance of lambs.

**Supplementary Information:**

The online version contains supplementary material available at 10.1186/s40104-025-01183-z.

## Background

As one of the essential nutrients for animals, dietary protein is intimately associated with the growth performance, nutritional metabolism, and immune regulation of the body [1]. However, excessive protein intake is considered as a potential threat to animal health and environmental protection [2]. Moreover, owing to the protein scarcity, reducing dietary protein levels might be the most effective nutritional approach to lower feed costs and nitrogen emissions [3]. Inevitably, the low-protein diet will decrease the productivity of animals, thereby reducing economic benefits [4]. Since amino acids (AAs) constitute the basic unit of proteins, therefore supplementing AAs appears to be a preferred and useful strategy to mitigate the adverse impacts associated with low dietary protein levels [5].

Methionine (Met) and lysine (Lys) are regarded as two of the most crucial limiting AAs for ruminants, and their incorporation into diets has been demonstrated to enhance animal health and production [3]. However, free AAs added directly to the diet are inevitably deaminated in the rumen by a variety of microorganisms, remarkably reducing the amount of essential AAs available for intestinal absorption [5]. On this premise, rumen-protected methionine (RPMet) and rumen-protected lysine (RPLys) and with an encapsulated layer are often used as additives to ensure that sufficient AAs reach the intestines in ruminants [3]. Previous research showed that the supplementation of 4.5 g/d RPLys plus 1.5 g/d RPMet to a low-protein diet (10.1% CP) increased daily weight gain and feed conversion in sheep [6]. In addition, studies in lambs showed that inclusion of RPMet [0.253 g/kg DM (dry matter)] and RPLys (0.50 g/kg DM) to a low-protein diet (12.5% CP) altered fat deposition through modulations of lipogenesis and lipolysis in the liver and muscle, and this may be associated with the modification of m^6^A RNA methylation [7]. However, in dairy sheep, addition of 2.5 g/kg RPMet (DM basis) to the diet exerted no beneficial effect on milk production and did not elicit changes in fatty acid profiles [8]. The discrepancy in observed outcomes may be attributed to variations in dietary regimes (additive amount, protein level, metabolic protein profile, energy nitrogen balance) and host factors (species, age, sex, health status) [9].

Protein metabolism in the rumen is the result of metabolic activity of ruminal microorganisms [10], however, greater attention should be paid to the importance of intestinal microbiome when rumen-protected AAs are supplemented in the diet. Rumen-undegradable protein, ruminal microbial protein, and rumen protected AAs constitute of the main sources of AAs in the intestine [11], where they will encounter with a variety of commensal microorganisms, in particular the hindgut [12, 13]. The colonic microorganisms are capable of metabolizing dietary protein and non-protein nitrogen in ruminants, thereby affecting nitrogen utilization efficiency [14]. Pioneer research demonstrated that RPLys inclusion improved amino acid balance and nitrogen utilization in dairy cows, which was partially associated with the enrichment of probiotic members *Christensenellaceae_R-7_group* and *Acinetobacter*, and the reduction of pathogenic members *Clostridium_sensu_stricto_1* and *Turicibacter* in the colon [15]. Despite these efforts, how the hindgut microbiota functions as a crucial element in maintaining the AA balance in response to rumen protected AA inclusion, thereby safeguarding animal functionality, warrant further exploration.

To fill the knowledge gap, we conducted an experiment with reducing dietary CP level of lambs and intervening with RPMet and RPLys supplementation. It is hypothesized that the inclusion of rumen-protected AAs to the low-protein diet can enhance animal growth through improving the AA balance and microbial metabolism in the hindgut. The colonic microbial compositions and functional potentials were depicted using metagenomic profiling, with emphasis on AA metabolism pathways.

## Materials and methods

### Animal ethics statement

All the procedures of this study were carried out following the guidelines approved by Animal Care and Use Committee of the Institute of Subtropical Agriculture, Chinese Academy of Sciences (permission No. ISA-R-2023-01).

### Animals and experimental design

Twenty-four healthy male Hulunbuir lambs (3.0 months old) with an initial weight of 18.5 ± 2.0 kg were selected in this study, and randomly allocated into one of three dietary treatments that lasted for 10 weeks (Table 1), with the first two weeks as the adaptation period. Experimental diets were formulated according to the feeding standard of meat-producing sheep and goats (China, NY/T816—2021 [16]). The control diet (CON) was formulated to have a crude protein (CP) content of 112.0 g/kg (DM basis), and the low-protein diet (LP) was formulated with a CP of 78.4 g/kg by reducing it by 30% compared to CON. The rumen-protected amino acid diet (LR) was the LP diet supplemented with 15 g/d RPMet and 10 g/d RPLys, based on our previous in vivo study in lambs and the company’s recommendation. The RPMet and RPLys products were manufactured with cooperation with a commercial company (Hangzhou King Techina Feed Co., Ltd., China), with 70% of lysine and 82% of methionine, respectively. Lambs were housed in individual pens, and fed twice daily at 08:00 and 16:00 in amounts to ensure less than 10% orts. All lambs had free access to water, and feed refusal was recorded daily. The body weight was measured at the beginning and end of the experiment.

**Table 1:** The ingredient and chemical composition of experimental diets (DM basis)

Item	Treatments^1^		
	**CON**	**LP**	**LR**
Ingredients, %			
Alfalfa	25.00	15.00	15.00
Oat grass	10.00	20.00	20.00
Corn	40.60	50.20	50.20
Wheat bran	7.60	10.60	10.60
Soybean meal	12.00	–	–
Fat powder	0.60	–	–
CaCO_3_	0.50	0.50	0.50
CaHPO_4_	1.00	1.00	1.00
Premix^2^	2.00	2.00	2.00
NaCl	0.50	0.50	0.50
MgO	0.20	0.20	0.20
Rumen-protected lysine, g/d	–	–	10.00
Rumen-protected methionine, g/d	–	–	15.00
Nutrient levels^3^, %			
DM	94.90	94.90	94.90
CP	11.20	7.84	7.84
Starch	33.93	41.67	41.67
NDF	35.65	40.13	40.13
ADF	18.03	17.97	17.97
GE, MJ/kg	17.26	17.81	17.81
Lys	0.70	0.38	1.20
Met	0.23	0.19	1.63

*DM *Dry matter, *CP *Crude protein, *NDF *Neutral detergent fiber, *ADF *Acid detergent fiber, *GE *Gross energy

^1^CON = control diet with crude protein of 112.0 g/kg; LP = low-protein diet with crude protein of 78.4 g/kg; LR = LP + 15 g/d RPMet and 10 g/d RPLys

^2^Supplied the following per kilogram premix: vitamin A 100,000 IU, vitamin D 50,000 IU, vitamin E 2,000 IU, Fe 2,000 mg, Cu 400 mg, Zn 5,000 mg, Mn 5,000 mg, I 100 mg, Co 10 mg, Se 10 mg

^3^Nutrient levels were measured values, the Lys and Met concentrations of the diets were calculated values

### Sample collection

The fecal samples from each lamb were collected through the floor drain type fecal collector for 7 consecutive days between d 62 to 68, and mixed to be a homogeneous sample. Separate steel funnels were used during collection to ensure that feces did not fall to the ground. Simultaneously, individual bags were used to collect the feces from each lamb to avoid cross contamination. Lambs were slaughtered at the end of the experiment. After slaughter, 1 cm colonic tissue of middle region was fixed in the 4% paraformaldehyde solution for morphologic analysis. Approximately 2 g of colonic content samples were collected from middle region, immediately frozen in liquid nitrogen, and stored at −80 °C prior to further microbial analysis [12]. Meanwhile, 2 g of colonic content samples were homogenized with 1 mL of 25% (w/v) metaphosphoric acid and 6 mL of water and then centrifuged (17,000 × *g* at 4 °C for 10 min), and the supernatant was stored at −20 °C for analysis of volatile fatty acids (VFAs) and ammonia nitrogen (NH_3_-N) [17]. Furthermore, 0.5 g of colonic content samples were homogenized with 1 mL of phosphate buffered saline (pH = 7.4), fully extracted at 4 °C for 12 h, and then centrifuged at 15,000 × *g* at 4 °C for 10 min. The supernatant was stored at −20 °C for analysis of free amino acids.

### Chemical analysis

The feed and fecal samples were dried at 65 °C for 72 h, and then grounded into powder by a disintegrator (ZX-1000Y, Taihe Industry and Trade Co., Ltd., China). Acid-insoluble ash was used as an internal marker to measure nutrient apparent total-tract digestibility and determined in a muffle furnace at 550 °C for 8 h [18]. Dry matter (method 934.01) were determined according to the Association of Official Analytical Chemists procedures (2000) [19]. According to the method 984.13 [19], the CP content was calculated based on the nitrogen concentration that determined with a flow injection apparatus (AA3, Seal Analytical, Germany). The acid-detergent fiber (ADF) and neutral detergent fiber (NDF) were analyzed by a fiber analyzer (FT12, Gerhardt, Germany) according to Van Soest et al. [20]. The starch content was determined by using the starch content assay kit (BC0705, Beijing Solarbio Technology Co., Ltd., China) according to the manufacturer's instructions.

### Colon morphology

Fixed colonic tissues were dehydrated and embedded in paraffin. Tissue sections (thickness of 4 μm) were stained with hematoxylin and eosin (HE, Olympus G1005; Wuhan Servicebio technology Co., Ltd., China), as detailed in previous study [12]. The mucosal thickness and muscle layer was measured using a fluorescence microscope (BX51, Olympus, Japan).

### Colonic microbial metabolites

Profiling of VFAs in the colonic content was conducted by calibration with the external standards of acetate, propionate, butyrate, isobutyrate, valerate, and isovalerate, using a gas chromatograph (7890A, Agilent, USA), according to the method of Jiao et al. [17]. The NH_3_-N concentration was determined with the phenol-hypochlorite method via a multifunctional enzyme labeling instrument (Infinite M200 PRO, TECAN, Switzerland) at a wavelength of 625 nm as described by Jiao et al. [21].

Determination of free amino acid concentrations in the colonic contents was conducted following the method by Wu et al. [13]. Briefly, 1 mL of the extracted supernatant was fully mixed with 1 mL of 8% sulfosalicylic acid solution, incubated at 4 °C for 12 h, and then centrifuged at 14,000 × *g* at 4 °C for 10 min. Afterwards, 1 mL of the supernatant was passed through the 0.22-μm polyether sulfone filter membrane, and determined by the fully automated amino acid analyzer (L8900, Hitachi, Japan).

### Quantification of colonic microbial protein and bacterial copy number

The microbial crude protein (MCP) content in the colonic digesta was determined using the purine method as detailed by Wu et al. [13]. Furthermore, microbial DNA extraction was conducted via the bead-beating method as detailed previously [13]. The DNA yield and integrity were assessed using a Qubit 2.0 Fluorometer (Thermo Scientific, MA, USA). Subsequently, absolute quantitative real-time PCR (qPCR) was performed to determine the copy numbers of the 16S rRNA genes of total bacteria, as detailed in our previous study [17]. The numbers were converted to log10 for further statistical analysis.

### Metagenomic analysis

Sequencing libraries were prepared with extracted microbial DNA using the BGI Optimal DNA Library Prep Kit (BGI, Shenzhen, China) following the manufacturer’s instructions, and metagenomic sequencing was performed using the 150 bp paired-end DNBSEQ T7 platform. Raw data was filtered using Fastp (v 0.21.0) to obtain clean data [22]. Clean reads aligned to the reference sheep genome (*Ovis aries*, GCA_011170295.1_ASM1117029v1) using BWA (v 0.7.17) were removed [23]. These high-quality non-host reads were subsequently de novo assembled separately using MEGAHIT (v 1.1.3) [24]. Contigs were annotated using Prodigal (v 2.6.3) software to predict open reading frames (ORFs) [25]. The non-redundant gene catalogue was constructed by using MMseqs2 (v 11-e1a1c) [26]. Non-host reads of each sample were mapped to the gene catalogue with 95% identity using BWA (v 0.7.17) [23]. Representative sequences of the gene catalog were searched against the NCBI NR database using MEGAN Community Edition (v 6.25.9) for taxonomic annotations [27]. Kyoto Encyclopedia of Genes and Genomes (KEGG) annotation was conducted against the KEGG database using KOBAS (v 3.0) [28]. Generalized Reporter Score-based Analysis (GRSA) was used to perform enrichment analysis based on the annotation results [29].

Contigs longer than 1.0 kb were used for binning into metagenome-assembled genomes (MAGs) using metaWRAP (v 1.2) with default parameters [30]. The completeness and contamination of the MAGs were estimated with CheckM (v 1.0.12) [31]. High-quality MAGs were refined according to the thresholds of ≥ 90% completeness and ≤ 5% contamination [32], and then dereplicated with a 95% ANI cutoff using dRep [33]. Consequently, 626 nonredundant high-quality MAGs (Table S4) were obtained. The MAGs were taxonomically annotated using GTDB-Tk (v 2.3.0) based on the Genome Taxonomy Database (v R214) [34].

### Statistical analysis

All the statistical analysis were conducted using R software (v 2.6.4). Data of growth performance, nutrient digestibility, and microbial metabolites were analyzed using one-way ANOVA followed by the post hoc Tukey’s HSD test. Analyses of the alpha and beta diversities of taxonomic and functional profiles of the colonic microbiome were performed using the vegan package [35]. The adonis function was used to implement PERMANOVA analysis of beta diversity, with Bray–Curtis distance matrix and 999 permutation tests. Furthermore, comparisons of microbial species, genes, and KOs were performed using the Kruskal–Wallis test. The *P*-values were adjusted using the false discovery rate (FDR) correction, and a *P*-value < 0.05 was regarded as statistically significant.

## Results

### Growth performance and nutrient digestibility

As shown in Table 2, despite that dry matter intake was similar (*P* > 0.05) among three groups, average daily weight gain (ADG) was lower in the LP group when compared to the CON and LR groups (*P* = 0.012), and it tender to be lower for LR vs. CON (*P* = 0.062). Accordingly, LP treatment tended to decrease feed conversion ratio when compared to the CON group (*P* = 0.097). In addition, the digestibility of dry matter and ADF tended to be greater in lambs fed the CON diet (*P* < 0.10). The LP treatment remarkably decreased CP digestibility (*P* < 0.001), while addition of RPMet and RPLys showed a slight improvement in CP digestibility. These results indicated that reducing CP content by 30% decreased animal growth, while inclusion of RPMet and RPLys to the LP diet tended to elicit a remission in weight gain and protein utilization efficiency.

**Table 2:** Effects of a low-protein diet supplemented with rumen-protected amino acids on growth performance and nutrient digestibility of lambs

Item	Treatments^1^			SEM	*P*-value
	**CON**	**LP**	**LR**		
Growth performance					
Dry matter intake, g/d	864.79	853.36	852.76	8.634	0.827
Average daily weight gain, g/d	155.81^a^	133.56^b^	143.31^ab^	3.329	0.012
Feed conversion ratio	5.66	6.43	5.95	0.152	0.097
Nutrient digestibility					
Dry matter digestibility, %	77.02	74.21	74.53	0.557	0.072
Crude protein digestibility, %	72.85^a^	54.95^b^	57.33^b^	1.930	< 0.001
NDF digestibility, %	73.26	69.75	69.31	1.008	0.221
ADF digestibility, %	68.13	62.65	62.69	1.113	0.085
Starch digestibility, %	95.24	94.63	94.27	0.530	0.769

^1^CON = control diet with crude protein of 112.0 g/kg; LP = low-protein diet with crude protein of 78.4 g/kg; LR = LP + 15 g/d RPMet and 10 g/d RPLys

^a,b^Values within a row with different superscripts differ significantly at *P* < 0.05

*NDF* Neutral detergent fiber, *ADF *Acid detergent fiber

### Colon morphology and microbial metabolites

**Fig. 1:**
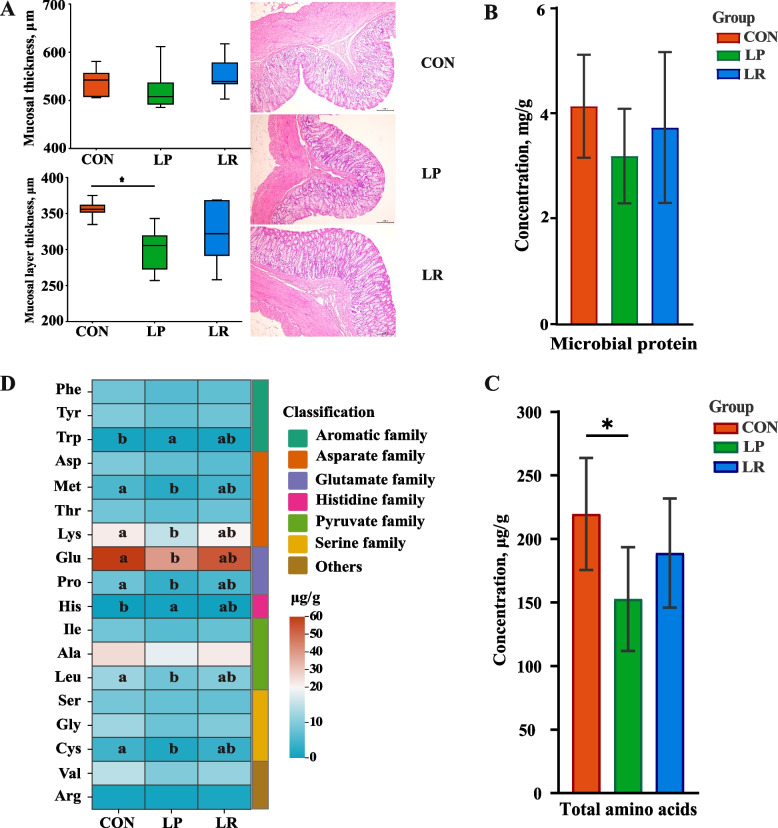
Effects of a low-protein diet supplemented with rumen-protected amino acids on morphological changes and microbial metabolites in the colon of lambs. **A** Mucosal thickness and muscle layer thickness. **B **The concentration of microbial protein. **C** The concentration of total amino acid. **D** Free amino acid profile. ^*^P < 0.05. ^a,b^Different letters in the same row indicate significant differences at *P* < 0.05

The mucosal thickness of the colon was similar among the three groups (*P* > 0.05), whereas the muscle layer was lower for the LP group when compared to the CON group (*P* < 0.05, Fig. 1A). In the colonic content, the NH_3_-N concentration was lower in the LP when compared to two other groups (*P* < 0.05, Table 3), while total VFA levels and MCP concentrations were similar among three groups (*P* > 0.05, Fig. 1B). The butyrate molar concentration was greater (*P* < 0.05), while acetate molar concentration (*P* < 0.05) was lower for lambs fed the LP and LR diets when compared to those fed the CON diet. Moreover, inclusion of RPMet and RPLys remarkably increase the molar concentrations of isobutyrate and isovalerate (*P* < 0.05). In terms of amino acid profile, the LP treatment remarkably decreased total AA concentration (*P* < 0.05, Fig. 1C), while inclusion of RPMet and RPLys showed an improvement in AA concentration (*P* < 0.05), which also applies to the change law of the concentrations of methionine, lysine, leucine, glutamate, cysteine, and proline (Fig. 1D). Meanwhile, the concentrations of tryptophan and histidine in the LP group were significantly higher compared with the CON group (*P* < 0.05), and their concentrations tended to be greater for LR compared to CON group.

**Table 3:** Effects of a low-protein diet supplemented with rumen-protected amino acids on fermentation characteristics in colon of lambs

Item	Treatments^1^			SEM	*P*-value
	**CON**	**LP**	**LR**		
Ammonia nitrogen, mmol/kg	2.89^b^	1.81^c^	3.80^a^	0.205	< 0.001
Total VFA, mmol/kg	54.50	56.32	54.84	1.698	0.905
VFA molar percentage, mol/100 mol					
Acetate	78.83^a^	75.24^b^	75.24^b^	0.610	0.023
Propionate	13.92	14.38	13.84	0.472	0.898
Isobutyrate	0.85^ab^	0.57^b^	1.07^a^	0.071	0.009
Butyrate	4.87^b^	8.44^a^	7.74^ab^	0.535	0.018
Isovalerate	0.61^ab^	0.38^b^	0.97^a^	0.076	0.004
Valerate	1.15^a^	0.89^b^	1.06^ab^	0.071	0.009

^1^CON = control diet with crude protein of 112.0 g/kg; LP = low-protein diet with crude protein of 78.4 g/kg; LR = LP + 15 g/d RPMet and 10 g/d RPLys

^a,b^Values with a row with different superscripts differ significantly at *P* < 0.05

*VFA *Volatile fatty acid

### Functional metabolic potentials of the colonic microbiome

**Fig. 2:**
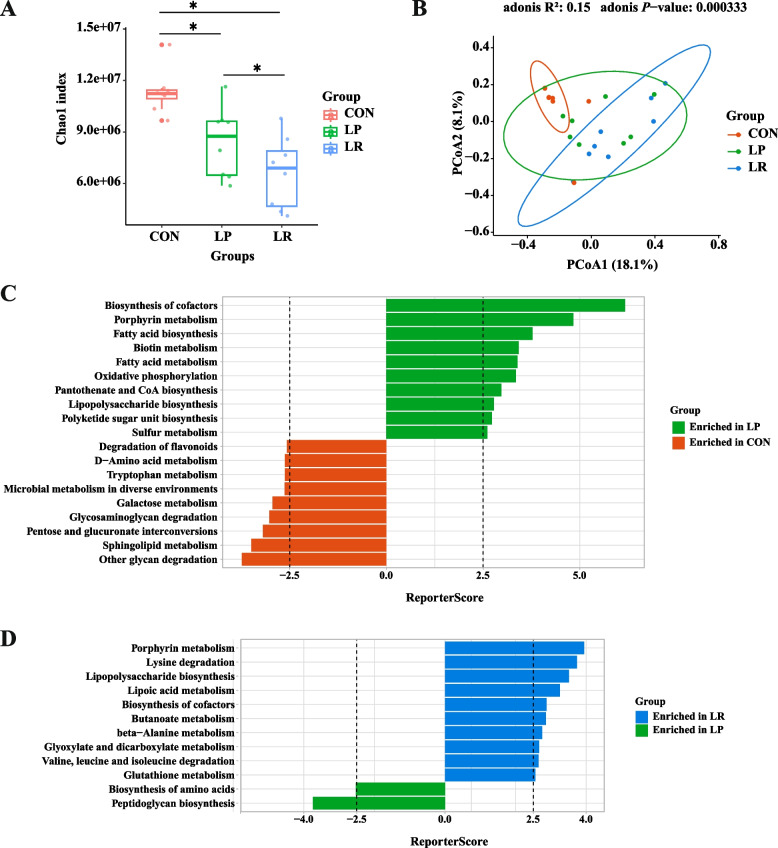
Effects of a low-protein diet supplemented with rumen-protected amino acids on microbial functional potentials in the colon of lambs. **A** Chao1 index based on gene profile. **B** Principal coordinate analysis (PCoA) based on Bray–Curtis dissimilarity of gene profile. **C**–**D** Differential KEGG pathways based on GRSA enrichment analysis of LP vs. CON (**C**) and LR vs. LP (**D**)

Metagenomes were sequenced to further understand the microorganisms and processes controlling the observed differences in colon metabolism (Table S1). Alpha diversity as measured by the Chao1 index at the gene level was dramatically decreased by LP and LR treatments in comparison to the CON group (*P* < 0.05, Fig. 2A). The PCoA analysis indicated that the functional potentials of colonic microbiome substantially changed with three dietary treatments (*P* < 0.05, Fig. 2B). The GRSA enrichment results showed the KEGG pathways including D-amino acid metabolism, tryptophan metabolism, and galactose metabolism were enriched in the LP treatment, while biosynthesis of cofactors, fatty acid biosynthesis and metabolism, oxidative phosphorylation, pantothenate and CoA biosynthesis were enriched in the CON treatment (Fig. 2C). Concurrently, LP treatment enriched biosynthesis of amino acids and peptidoglycan biosynthesis pathways, inclusion of RPMet and RPLys to the LP diet enriched for pathways including biosynthesis of cofactors, beta-alanine metabolism, and valine, leucine and isoleucine degradation (Fig. 2D). Hence, it is reasonable to infer the microbial metabolic potentials referring to VFA biosynthesis and AA metabolism was remarkably altered by three dietary treatments.

### Microbial VFA biosynthesis and AA metabolism in the colon

**Fig. 3:**
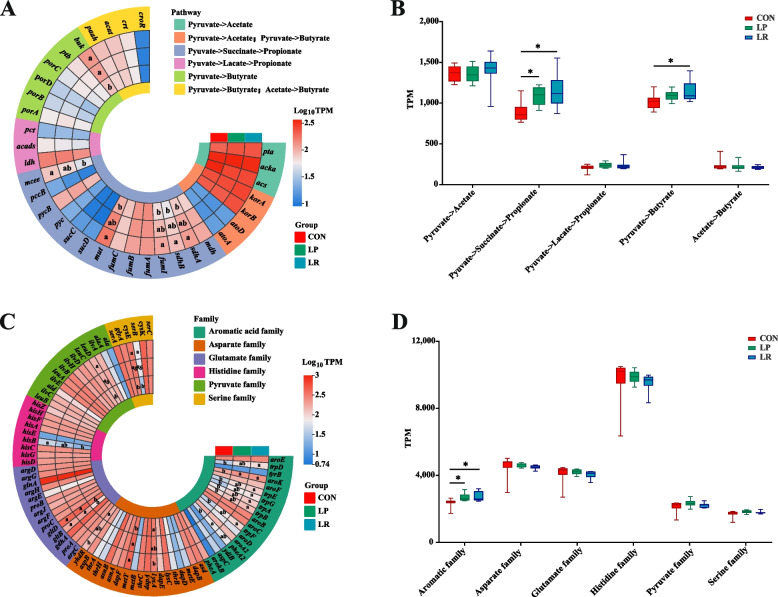
Effects of a low-protein diet supplemented with rumen-protected amino acids on microbial volatile fatty acid and amino acid production potentials in the colon of lambs. **A** and **B** The abundance of KOs related to the synthesis and metabolism of volatile fatty acids in the colon. **C** and **D** The abundance of KOs related to the biosynthesis of amino acids in the colon. ^*^P < 0.05. ^a,b^Different letters in the same sector indicate significant differences at *P* < 0.05

Give the significant differences observed in VFA and AA profiles, we screened for the genes encoding for enzymes implicated in metabolic cascades of VFA biosynthesis and AA metabolism of colonic microorganisms (Tables S2 and S3). Intriguingly, in term of VFA biosynthesis, the LP and LR treatment enriched for the propionate production via succinate as the intermediate (*sdhA*, *sdhB*, *fumA*, *MUT*, and *epi* genes), pyruvate to butyrate production (*buk* gene) (*P* < 0.05; Fig. 3A and B).

We further analyzed signature genes that support biosynthesis of AAs, which were organized into six major families, reflecting the diverse biosynthetic pathways through which GIT microorganisms produce them [36]. Intriguingly, the abundance of signature genes for lysine biosynthesis (*lysA* and *dapF* genes) were lower, while the abundance of signature genes for methionine biosynthesis (*metB* and *metY* genes) were greater for the LR treatment when compared to those for the CON treatment (*P* < 0.05, Fig. 3C and D). Furthermore, the abundances of signature genes related to the synthesis of tryptophan (*trpD*, *trpE*, *trpG*, *trpA*, *trpB*, and *trpF* genes) and glutamate (*gltB* and *gltD* genes) were remarkably greater for LP and LR treatment in comparison to the CON treatment (*P* < 0.05). In addition, the abundances of signature genes related to the synthesis of pyruvate family AAs, including leucine (*leuB* and *leuD* genes) and isoleucine (*ilvG* gene) were greater for the LP treatment when compared to those in the CON group (*P* < 0.05). Altogether, these results suggest that these three diets differentially influence VFA and AA production, likely due to the promotion of distinct microbial metabolic pathways in the colon.

### Microbial diversity and composition in the colon based on MAGs

**Fig. 4:**
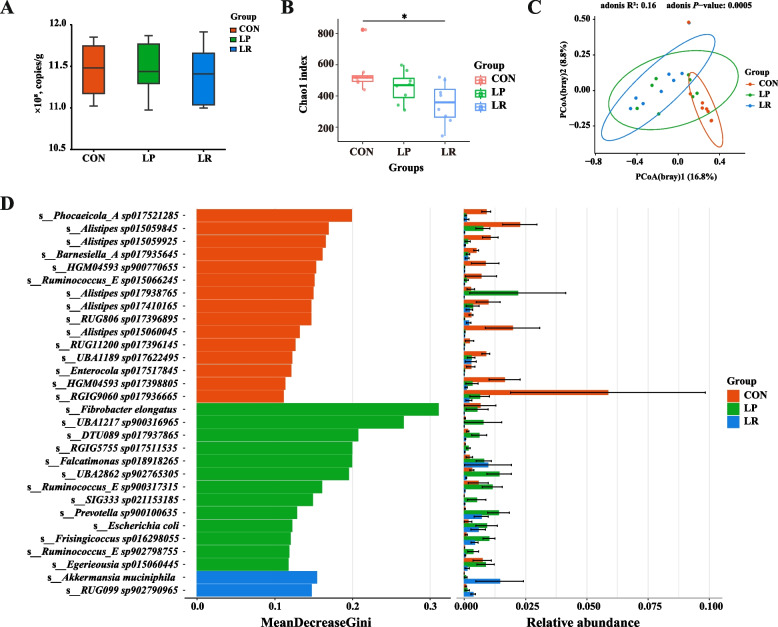
Effects of a low-protein diet supplemented with rumen-protected amino acids on microbial diversity and structure based on MAGs in the colon of lambs. **A** Copy numbers of the 16S rRNA genes of total bacteria. **B** Chao1 index at the MAG level. **C** Principal coordinate analysis (PCoA) based on Bray–Curtis dissimilarity of microbial composition at the MAG level. **D** Random forest results for colonic microbiota at species level. ^*^P < 0.05

As indicated by qPCR results, total bacteria copy number was not affected by the three dietary treatments (*P* > 0.05, Fig. 4A). Meanwhile, we conducted a metagenomic binning analysis of these 24 colonic samples, and recovered 626 high-quality MAGs with the thresholds of ≥ 90% completeness and ≤ 5% contamination. Taxonomic analysis of these microbial consortia based on Genome Taxonomy Database revealed the presence of 15 phyla, 21 classes, 37 orders, 75 families, 234 genera and 293 species (Table S4).

Notably, alpha diversity as measured by the Chao1 index at the MAG level was greater in the CON treatment when compared to the LR treatment (*P* < 0.05, Fig. 4B). The PCoA analysis based on the Bray–Curtis distance at the MAG level indicated that each dietary treatment exhibited its distinct microbiome (*P* < 0.05, Fig. 4C). Insights from random forest analysis identified 30 microbial biomarkers for three dietary treatments (Fig. 4D). Of note, *Phocaeicola_A sp017521285*, *Alistipes_sp015059845*, *Barnesiella_A sp017935645*, and *Ruminococcus_E sp015066245* were selected as featured biomarkers in the CON treatment; *Fibrobacter_elongatus*, *UBA1217 sp900316965*, *Prevotella sp900100635*, *Ruminococcus_E sp900317315,* and *Escherichia coli* were enriched in the LP treatment; and *Akkermansia muciniphila* and *RUG099 sp902790965* were featured biomarker in LR treatment.

## Discussion

Feeding a LP diet to ruminant animals is a generally accepted nutritional strategy to reduce the feed cost and lower environmental pollution, during which it is critical to balance the supply of metabolizable AAs in the diets [37, 38]. In the current study, RPMet and RPLys were supplemented in the rumen-protected form in lambs during LP challenge for ultimate productive performance. As anticipated, their inclusion results in increments for weight gain, similar to previous observations in dairy cows and sheep [6, 38]. Specifically, the addition of 4.5 g/d RPLys plus 1.5 g/d RPMet resulted in a 40% increase in ADG in Tan lambs [6], and feeding RPLys at 8.5% of metabolic protein tended to increase milk protein yield and body weight gain by 0.07 kg/d and 0.09 kg/d in dairy cows, respectively [38]. Of note, addition of RPMet and RPLys showed a slight improvement in the reduced CP digestibility induced by LP challenge. This might be attributed to discrepancy in dietary protein sources, with low-quality corn and wheat bran in the LP diet, whereas high-quality soybean meal in the CON diet. Generally, soybean meal, as a high-quality protein source characterized by a relatively loose protein structure, exhibited 7.6 times and 2.8 times greater digestible protein than the corn and wheat bran [16, 39]. Concurrently, RPMet and RPLys supplementation improved the intestinal amino acid supply, thereafter elicited the promotion in animal growth [40].

The integrity of the intestinal mucosa and epithelial tissue structure is crucial for animals to effectively absorb and utilize nutrients [41]. The thicker mucosal layer in the CON treatment led to an increase in the surface area in contact with the chyme, and thereafter facilitated nutrient transport in the colon [41]. It is widely accepted that the gut microbiota serve as a crucial component in maintaining nutrient metabolism and intestinal immunity of the host, through their microbial metabolites such as VFAs, NH_3_-N, and AAs [42]. The colon, characterized by its high microbial abundance, is widely regarded as the terminal point for nutrient absorption in the gastrointestinal tract of ruminants [12]. Colonic fermentation has been demonstrated to play a pivotal role in energy supply and nitrogen metabolism, and optimizing colonic fermentation has been shown to enhance nutrient utilization efficiency [13, 14]. As anticipated, the three dietary treatments remarkably altered microbial metabolite profiles in the colon. Firstly, the LP and LR treatments increased butyrate production in comparison to CON treatment, which could be directly utilized by epithelial cells to promote the development of the gastrointestinal tract and thereafter regulating the whole-body energy homeostasis and metabolic function balance [43]. Secondly, during the LP challenge, the LR treatment promoted the production of branched-chain fatty acids (BCFAs) and NH_3_-N. This can be attributed to the dissolution and release of RPAA in the colon, with a large quantity of methionine and lysine furnishing more amino donors to microbiota [44]. Finally, as expected, the LP treatment remarkably decreased the concentrations of methionine, lysine, and leucine, while inclusion of RPMet and RPLys showed an improvement. As limiting AAs in ruminants, methionine plays a significant role in cellular methylation reactions and redox maintenance [45], and lysine is involved in regulating cellular metabolic pathways [46]. Furthermore, the improvement of leucine by RPAA addition might be linked to the increased levels of BCFAs, and thereafter regulating glucose homeostasis and energy metabolism balance in the body through activating the mechanical target of rapamycin complex 1 (mTORC1) [47]. Intriguingly, LP and LR treatments showed substantial improvement in tryptophan concentration, whose catabolites affects various physiological processes and may contribute to intestinal and systemic homeostasis in health and disease [48]. Collectively, the addition of RPMet and RPLys under low protein diet levels enhanced butyrate production and balanced amino acid profile, which exerts beneficial effects for the growth of lambs.

Metagenomic insights uncovered the microbial metabolism pathways responsible for the production of diverse metabolites during three dietary interventions. It is noteworthy that genes involved in the pyruvate conversion to propionate through succinate, such as succinate dehydrogenase (*sdh*), fumarate hydratase (*fum*), and methyl malonyl-CoA mutase (*mut*), were enriched under LR treatment. Despite the fact that propionate molar proportion was similar among three dietary treatment, the intermediate succinate is involved in the tricarboxylic acid cycle (TCA cycle) [49], and affecting the VFA profile. Not surprisingly, the LP and LR treatments elicited an increase in the abundance of butyrate kinase (*buk*) gene, signifying a higher potential for producing butyrate. This might be attributed to the greater NDF and starch content, and the colonic microbiota, particularly members of Clostridia and Bacteroides, possess the capacity to covert resistant starch and non-starch polysaccharides into butyrate [50]. More importantly, the encapsulated RPLys could be released as lysine in the colon, which can be converted into branched chain amino acids (BCAAs), which subsequently give rise to BCFAs [51]. Correspondingly, valine, leucine and isoleucine degradation pathway was enriched in LR treatment, partially implicated in the elevated levels of leucine and BCFAs. Moreover, glutamate synthase (*glt*) is involved in the preceding steps of ammonia assimilation and biosynthesis of other amino acids, which transfers the amino group to alpha ketoglutaric acid [52]. Hence, the increase of both total AAs and glutamate in LR treatment may prove the de novo synthesis of other AAs. Finally, the abundances of most genes implicated in tryptophan biosynthesis were enriched in the LR treatment [47], which coincides with the elevated tryptophan concertation observed above. Undoubtedly, the supplementation of RPMet and RPLys improved microbial VFA and AA metabolic potentials during LP challenge in the colon of lambs.

Metagenome binning can capture substantial microbial diversity through direct analysis of genetic information, and subsequently generate reference genomes, which are essential resources for understanding the functional role of individual microorganisms and identifying novel microbial lineages [12]. In this study, species-level microbial biomarkers selected by three dietary treatments were identified through random forest analysis of the 626 recovered high-quality MAGs. Of particular interest, the CON diet is featured by enrichment of *Alistipes* spp., *Phocaeicola* spp., and *Ruminococcus* spp. *Alistipes* and *Phocaeicola* are main acetate producers in the gut [53, 54], and *Ruminococcus* spp. are renowned for its capacity to degrade cellulose and hemicellulose [55], all of which jointly contribute to the acetate accumulation in the CON group. Furthermore, the LP diet selects colonic *Fibrobacter elongatus*, *Prevotella* spp., *Ruminococcus* spp., and *Escherichia coli*. As typical fiber degraders, the former three microbial members contributed to the fermentation of greater level of dietary carbohydrates during LP challenge [56]. Nevertheless, the surge of *Escherichia coli*, active AA metabolizing bacteria, might contribute to the adverse effect of LP challenge and impair the intestinal environment and host health [36, 57]. Intriguingly, the interventions of RPMet and RPLys preferably enriched *Akkermansia muciniphila* in the colon, which has been reported to elicit positive impacts on metabolic diseases and intestinal barrier function [58]. Therefore, RPMet and RPLys demonstrate the potential to promote the construction of beneficial microbial communities in the colon.

## Conclusions

Adding 15 g/d of RPMet and 10 g/d of RPLys to a low-protein diet elicited a positive effect on the growth performance of lambs, and this was driven through dietary selection of colonic microbiota. The supplementation of RPMet and RPLys during LP challenge improved the VFA and AA biosynthesis potentials and preferably enriched the probiotic *Akkermansia muciniphila* in the colon microbiome.

## Supplementary Information

Additional file 1: Table S1. Sample information. Table S2. Genes and KOs for volatile fatty acid production. Table S3. Genes and KOs for amino acid production. Table S4. GTDB taxonomy of 626 MAGs.

## Data Availability

Raw sequence reads for all samples have been deposited into the China National GeneBank DataBase (CNGBdb) with accession number CNP0006433.

## Abbreviations

AA: Amino acid
ADF: Acid detergent fiber
ADG: Average daily weight gain
BCAA: Branched-chain amino acid
BCFA: Branched-chain fatty acid
CP: Crude protein
DM: Dry matter
GE: Gross energy
MAG: Metagenome-assembled genome
MCP: Microbial crude protein
NDF: Neutral detergent fiber
NH_3_-N: Ammonia nitrogen
RPLys: Rumen-protected lysine
RPMet: Rumen-protected methionine
VFA: Volatile fatty acid
